# A bi-TTF with a bipyridine spacer: 4,4′-bis­[(3,6,7-trimethyl­sulfanyltetra­thia­fulvalen-2-yl)sulfanylmeth­yl]-2,2′-bipyridine

**DOI:** 10.1107/S1600536808039111

**Published:** 2008-11-29

**Authors:** Lakhemici Kaboub, Jean-Marc Fabre, Jean-Pierre Legros

**Affiliations:** a Laboratoire de Chimie des Matériaux Organiques, Centre Universitaire de Tébessa, Route de Constantine, 12000 Tébessa, Algeria; b Institut Charles Gerhardt, UMR CNRS 5253, AM2N, ENSCM, 8 rue de l’Ecole Normale, F-34296 Montpellier cedex 5, France; c CNRS; LCC (Laboratoire de Chimie de Coordination), 205 route de Narbonne, F-31077 Toulouse, France; d Université de Toulouse; UPS,INPT; LCC, F-31077 Toulouse, France

## Abstract

The title compound, C_30_H_28_N_2_S_16_, is a precursor to hybrid magnetic materials. The complete molecule is generated by a crystallographic inversion centre. In the crystal structure, the TTF core is not planar and adopts a chair conformation; the two C_3_S_2_ rings are folded around the S⋯S hinges, the dihedral angles being 17.14 (8) and 13.46 (7)°. There is a short S⋯S contact [3.4863  (14) Å] in the crystal structure.

## Related literature

For general background, see: Yagubskii (1993 ▶); Williams *et al.* (1992 ▶); Sakata *et al.* (1998 ▶); Fabre (2002 ▶). For coordination complexes of TTF with nitro­gen aromatic substituents, see: Setifi *et al.* (2003 ▶); Liu *et al.* (2003 ▶); Boudiba *et al.* (2005 ▶). For the double Wittig coupling reaction used in the synthesis of the bi-TTF(bipyridine), see: Ikeda *et al.* (1993 ▶); Gonzales *et al.* (2000 ▶). For the synthesis of the precursors, see: Doria *et al.* (1986 ▶); Hudhomme *et al.* (2006 ▶); Blanchard *et al.* (1993 ▶).
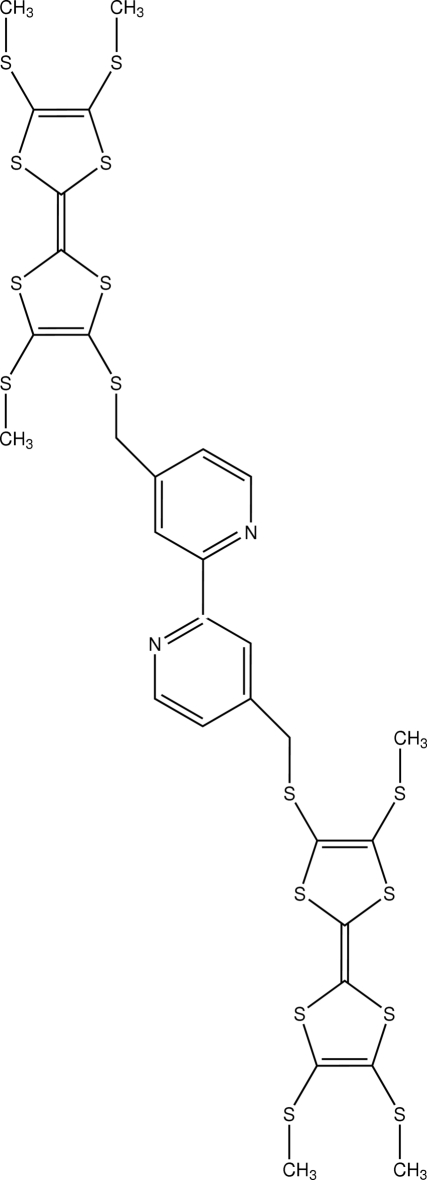


## Experimental

### 

#### Crystal data

C_30_H_28_N_2_S_16_
*M* *_r_* = 929.50
Triclinic, 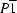

*a* = 7.4840 (12) Å
*b* = 7.7691 (11) Å
*c* = 17.707 (3) Å
α = 88.973 (12)°
β = 80.071 (13)°
γ = 72.245 (13)°
*V* = 965.2 (3) Å^3^
*Z* = 1
Mo *K*α radiation
μ = 0.92 mm^−1^
*T* = 293 (2) K
0.19 × 0.11 × 0.06 mm

#### Data collection

Oxford Diffraction XCalibur diffractometer with CCD detector
Absorption correction: multi-scan (Blessing, 1995 ▶) *T*_min_ = 0.884, *T*_max_ = 0.937
6690 measured reflections
3391 independent reflections
1942 reflections with *I* > 2σ(*I*)
*R*_int_ = 0.038

#### Refinement

*R*[*F* ^2^ > 2σ(*F* ^2^)] = 0.035
*wR*(*F* ^2^) = 0.070
*S* = 0.83
3391 reflections
220 parameters
H-atom parameters constrained
Δρ_max_ = 0.23 e Å^−3^
Δρ_min_ = −0.21 e Å^−3^

### 

Data collection: *CrysAlis CCD* (Oxford Diffraction, 2006 ▶); cell refinement: *CrysAlis RED* (Oxford Diffraction, 2006 ▶); data reduction: *CrysAlis RED*; program(s) used to solve structure: *SIR92* (Altomare *et al.*, 1993 ▶); program(s) used to refine structure: *SHELXL97* (Sheldrick, 2008 ▶); molecular graphics: *ORTEPIII* (Burnett & Johnson, 1996 ▶), *CAMERON* (Watkin *et al.*, 1993 ▶) and *ORTEP-3* (Farrugia, 1997 ▶); software used to prepare material for publication: *WinGX* (Farrugia, 1999 ▶).

## Supplementary Material

Crystal structure: contains datablocks I, global. DOI: 10.1107/S1600536808039111/nc2124sup1.cif

Structure factors: contains datablocks I. DOI: 10.1107/S1600536808039111/nc2124Isup2.hkl

Additional supplementary materials:  crystallographic information; 3D view; checkCIF report

## Figures and Tables

**Fig. 1.**
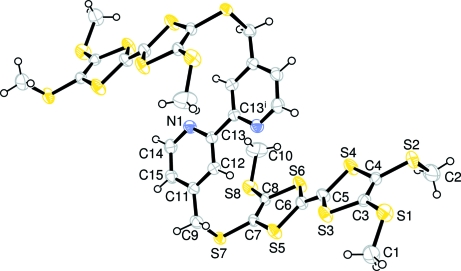
The molecular structure of the title compound, with atom labels and 50% probability displacement ellipsoids for non-H atoms. Symmetry code: i = -x+1, -y+1, -z+1.

## supplementary crystallographic information

### Comment

To date the search for solids presenting two physical properties such as magnetism and electrical conductivity inside a same material has expanded greatly, particularly with materials involving tetrathiafulvalene (TTF) derivatives well known to provide conducting and even superconducting salts (Williams *et al.*, 1992; Yagubskii,1993; Sakata *et al.*, 1998: Fabre, 2002). To introduce a magnetic network, involving localized spins, inside such conducting salts, a particularly promising way is to build a coordination complex between a transition metal (Cu, Co ···) and a pyridine or bipyridine moiety bonded to a TTF core (Setifi *et al.*, 2003; Liu *et al.*, 2003; Boudiba *et al.*, 2005). Following this strategy we synthesized the title precursor: bi-TTF(bipyridine) 1 and studied its crystal structure to verify if the molecular geometry could allow a subsequent easy formation of the target coordinating complex. The molecular structure is shown in Fig. 1. A s expected two TTF cores bearing methylsulfanyl substituents are connected by a bipyridine spacer. The molecule lies on a crystallographic centre of symmetry located at the centre of the bipyridine moiety, the asymmetric unit is thus composed of half a molecule. As a result the bipyridine spacer is in the *trans* conformation. The TTF cores deviate strongly from planarity and take a chair conformation. The two C_3_S_2_ rings are folded around the S···S hinges: the central group S3/S4/C5/C6/S5/S6 is planar and the external planes S3/S4/C3/C4 and S5/S6/C7/C8 make dihedral angles of 17.14 (8)° and 13.46 (7)° respectively. There is a short S&ctdot;S contact [3.4863 (14) Å] in the crystal structure.

### Experimental

The bi-TTF(bipyridine) 1 was synthesized (37% yield) by using a double Wittig coupling reaction (Ikeda, 1993*; *Gonzalez, 2000) between two appropriate formyl-TTF units and 4,4'-bis(methyltripenylphosphonium)-2,2'-bipyridinedibromide previously obtained as described in the literature (Doria, 1986). Red crystals (m.p.: 158°C) of 1 were obtained as thin platelets by slow evaporation of a solution of 1 in a mixture of dichloromethane-acetonitrile.

### Refinement

H atoms were located in a difference map then positioned geometrically and refined using a riding model with C—H distances set to 0.96 Å (*sp^3^*) and 0.93 Å (*sp^2^*), and *U*_iso_(H) egal to 1.2 times the equivalent *U*_iso _of the atom of attachment.

### Crystal data

**Table:**

C_30_H_28_N_2_S_16_	*Z* = 1
*M**_r_* = 929.50	*F*_000_ = 478
Triclinic, *P*1	*D*_x_ = 1.599 Mg m^−^^3^
Hall symbol: -P 1	Melting point: 431 K
*a* = 7.4840 (12) Å	Mo *K*α radiation λ = 0.71073 Å
*b* = 7.7691 (11) Å	Cell parameters from 1223 reflections
*c* = 17.707 (3) Å	θ = 2.9–25.0º
α = 88.973 (12)º	µ = 0.92 mm^−^^1^
β = 80.071 (13)º	*T* = 293 (2) K
γ = 72.245 (13)º	Block, red
*V* = 965.2 (3) Å^3^	0.19 × 0.11 × 0.06 mm

### Data collection

**Table:**

Oxford Diffraction XCalibur diffractometer with CCD detector	3391 independent reflections
Radiation source: fine-focus sealed tube	1942 reflections with *I* > 2σ(*I*)
Monochromator: graphite	*R*_int_ = 0.039
*T* = 293(2) K	θ_max_ = 25.0º
φ and ω scans	θ_min_ = 2.9º
Absorption correction: multi-scan(Blessing, 1995)	*h* = −8→8
*T*_min_ = 0.884, *T*_max_ = 0.937	*k* = −9→9
6690 measured reflections	*l* = −18→21

### Refinement

**Table:**

Refinement on *F*^2^	Secondary atom site location: difference Fourier map
Least-squares matrix: full	Hydrogen site location: inferred from neighbouring sites
*R*[*F*^2^ > 2σ(*F*^2^)] = 0.035	H-atom parameters constrained
*wR*(*F*^2^) = 0.071	*w* = 1/[σ^2^(*F*_o_^2^) + (0.0277*P*)^2^] where *P* = (*F*_o_^2^ + 2*F*_c_^2^)/3
*S* = 0.83	(Δ/σ)_max_ = 0.013
3391 reflections	Δρ_max_ = 0.23 e Å^−^^3^
220 parameters	Δρ_min_ = −0.21 e Å^−^^3^
Primary atom site location: structure-invariant direct methods	Extinction correction: none

### Special details

**Table:**

Experimental. Excalibur (Oxford Diffraction) four-circle Kappa geometry diffractometer equipped with an area CCD detector. Crystal-detector distance (mm): 70.0
Geometry. All e.s.d.'s (except the e.s.d. in the dihedral angle between two l.s. planes) are estimated using the full covariance matrix. The cell e.s.d.'s are taken into account individually in the estimation of e.s.d.'s in distances, angles and torsion angles; correlations between e.s.d.'s in cell parameters are only used when they are defined by crystal symmetry. An approximate (isotropic) treatment of cell e.s.d.'s is used for estimating e.s.d.'s involving l.s. planes.
Refinement. Refinement of *F*^2^ against ALL reflections. The weighted *R*-factor *wR* and goodness of fit *S* are based on *F*^2^, conventional *R*-factors *R* are based on *F*, with *F* set to zero for negative *F*^2^. The threshold expression of *F*^2^ > σ(*F*^2^) is used only for calculating *R*-factors(gt) *etc*. and is not relevant to the choice of reflections for refinement. *R*-factors based on *F*^2^ are statistically about twice as large as those based on *F*, and *R*-factors based on ALL data will be even larger.

### Fractional atomic coordinates and isotropic or equivalent isotropic displacement parameters (Å^2^)

**Table:**

	*x*	*y*	*z*	*U*_iso_*/*U*_eq_	
C1	−0.3976 (5)	0.7011 (5)	0.9533 (2)	0.0573 (11)	
H1A	−0.3692	0.6081	0.9142	0.069*	
H1B	−0.5251	0.7212	0.9806	0.069*	
H1C	−0.3095	0.6642	0.9884	0.069*	
C2	0.0146 (6)	1.2151 (5)	0.9481 (2)	0.0681 (13)	
H2A	−0.0135	1.1531	0.9938	0.082*	
H2B	−0.0070	1.3402	0.9609	0.082*	
H2C	0.1455	1.1612	0.9247	0.082*	
C3	−0.1411 (4)	0.8506 (4)	0.86485 (17)	0.0307 (8)	
C4	−0.0438 (4)	0.9702 (4)	0.85327 (17)	0.0300 (8)	
C5	0.1986 (4)	0.6686 (4)	0.79718 (18)	0.0339 (8)	
C6	0.3591 (4)	0.5317 (4)	0.77605 (17)	0.0325 (8)	
C7	0.6075 (4)	0.2257 (4)	0.72586 (16)	0.0271 (8)	
C8	0.7017 (4)	0.3475 (4)	0.71193 (17)	0.0277 (8)	
C9	0.5564 (5)	−0.0019 (4)	0.62192 (17)	0.0366 (8)	
H9A	0.5827	−0.1235	0.6028	0.044*	
H9B	0.4232	0.0432	0.6433	0.044*	
C10	0.9345 (5)	0.4866 (5)	0.6095 (2)	0.0711 (13)	
H10A	0.8469	0.4962	0.5748	0.085*	
H10B	1.0597	0.4718	0.5809	0.085*	
H10C	0.8944	0.5944	0.6417	0.085*	
C11	0.6020 (4)	0.1130 (4)	0.55723 (17)	0.0296 (8)	
C12	0.5025 (4)	0.2947 (4)	0.55804 (17)	0.0308 (8)	
H12	0.4020	0.3454	0.5979	0.037*	
C13	0.5516 (4)	0.4015 (4)	0.49995 (17)	0.0276 (8)	
C14	0.7883 (5)	0.1602 (4)	0.44029 (19)	0.0381 (9)	
H14	0.8869	0.1118	0.3995	0.046*	
C15	0.7491 (4)	0.0458 (4)	0.49646 (17)	0.0351 (8)	
H15	0.8209	−0.0757	0.4935	0.042*	
N1	0.6944 (4)	0.3353 (3)	0.44078 (14)	0.0343 (7)	
S1	−0.37725 (13)	0.90564 (13)	0.91017 (6)	0.0500 (3)	
S2	−0.13591 (13)	1.19803 (12)	0.88259 (5)	0.0442 (3)	
S3	−0.02471 (12)	0.63191 (11)	0.82438 (5)	0.0408 (2)	
S4	0.18635 (13)	0.89647 (12)	0.79792 (5)	0.0451 (3)	
S5	0.37241 (12)	0.30319 (11)	0.77697 (5)	0.0419 (3)	
S6	0.58066 (12)	0.56994 (11)	0.74683 (5)	0.0409 (3)	
S7	0.69659 (12)	−0.00222 (11)	0.69692 (5)	0.0333 (2)	
S8	0.93880 (12)	0.29726 (12)	0.66720 (5)	0.0453 (3)	

### Atomic displacement parameters (Å^2^)

**Table:**

	*U*^11^	*U*^22^	*U*^33^	*U*^12^	*U*^13^	*U*^23^
C1	0.051 (2)	0.054 (3)	0.064 (3)	−0.026 (2)	0.017 (2)	−0.008 (2)
C2	0.108 (4)	0.050 (3)	0.059 (3)	−0.032 (3)	−0.034 (3)	0.001 (2)
C3	0.0287 (19)	0.033 (2)	0.0275 (19)	−0.0055 (15)	−0.0035 (15)	0.0019 (15)
C4	0.0294 (19)	0.0278 (19)	0.0287 (19)	−0.0028 (15)	−0.0052 (15)	−0.0004 (15)
C5	0.0280 (19)	0.0282 (19)	0.045 (2)	−0.0107 (16)	−0.0004 (16)	0.0009 (16)
C6	0.0274 (19)	0.0291 (19)	0.038 (2)	−0.0093 (16)	0.0030 (16)	−0.0033 (16)
C7	0.0237 (18)	0.0235 (19)	0.0299 (19)	−0.0010 (14)	−0.0044 (15)	−0.0019 (14)
C8	0.0224 (18)	0.0268 (19)	0.0293 (18)	−0.0038 (15)	0.0014 (15)	−0.0034 (15)
C9	0.042 (2)	0.031 (2)	0.041 (2)	−0.0131 (16)	−0.0135 (18)	0.0027 (16)
C10	0.046 (3)	0.083 (3)	0.079 (3)	−0.027 (2)	0.011 (2)	0.025 (3)
C11	0.0317 (19)	0.0278 (19)	0.033 (2)	−0.0109 (15)	−0.0132 (16)	0.0020 (15)
C12	0.0281 (18)	0.033 (2)	0.0307 (19)	−0.0090 (15)	−0.0052 (15)	0.0016 (15)
C13	0.0264 (18)	0.0279 (18)	0.0290 (18)	−0.0063 (14)	−0.0098 (15)	0.0016 (15)
C14	0.031 (2)	0.038 (2)	0.041 (2)	−0.0062 (17)	−0.0012 (17)	−0.0046 (17)
C15	0.036 (2)	0.0276 (19)	0.040 (2)	−0.0027 (16)	−0.0142 (18)	−0.0004 (17)
N1	0.0322 (16)	0.0298 (17)	0.0366 (17)	−0.0053 (13)	−0.0019 (14)	0.0005 (13)
S1	0.0310 (5)	0.0449 (6)	0.0615 (7)	−0.0045 (4)	0.0131 (5)	−0.0036 (5)
S2	0.0453 (6)	0.0301 (5)	0.0517 (6)	−0.0035 (4)	−0.0073 (5)	−0.0098 (4)
S3	0.0268 (5)	0.0278 (5)	0.0627 (6)	−0.0086 (4)	0.0076 (5)	−0.0077 (4)
S4	0.0335 (5)	0.0282 (5)	0.0667 (7)	−0.0098 (4)	0.0105 (5)	−0.0042 (5)
S5	0.0310 (5)	0.0280 (5)	0.0620 (7)	−0.0109 (4)	0.0086 (5)	−0.0017 (4)
S6	0.0281 (5)	0.0266 (5)	0.0636 (7)	−0.0094 (4)	0.0061 (5)	−0.0077 (4)
S7	0.0382 (5)	0.0230 (5)	0.0360 (5)	−0.0030 (4)	−0.0107 (4)	0.0023 (4)
S8	0.0246 (5)	0.0390 (5)	0.0627 (7)	−0.0041 (4)	0.0075 (5)	−0.0003 (5)

### Geometric parameters (Å, °)

**Table:**

C1—S1	1.788 (3)	C8—S8	1.741 (3)
C1—H1A	0.9599	C8—S6	1.755 (3)
C1—H1B	0.9599	C9—C11	1.497 (4)
C1—H1C	0.9599	C9—S7	1.828 (3)
C2—S2	1.783 (4)	C9—H9A	0.9600
C2—H2A	0.9599	C9—H9B	0.9600
C2—H2B	0.9599	C10—S8	1.771 (3)
C2—H2C	0.9599	C10—H10A	0.9599
C3—C4	1.338 (4)	C10—H10B	0.9599
C3—S1	1.736 (3)	C10—H10C	0.9599
C3—S3	1.756 (3)	C11—C12	1.380 (4)
C4—S2	1.744 (3)	C11—C15	1.380 (4)
C4—S4	1.758 (3)	C12—C13	1.381 (4)
C5—C6	1.340 (4)	C12—H12	0.9300
C5—S4	1.744 (3)	C13—N1	1.342 (4)
C5—S3	1.765 (3)	C13—C13^i^	1.489 (6)
C6—S5	1.747 (3)	C14—N1	1.327 (4)
C6—S6	1.762 (3)	C14—C15	1.376 (4)
C7—C8	1.339 (4)	C14—H14	0.9300
C7—S7	1.743 (3)	C15—H15	0.9300
C7—S5	1.761 (3)		
			
S1—C1—H1A	109.5	C11—C9—H9B	109.4
S1—C1—H1B	109.5	S7—C9—H9B	109.4
H1A—C1—H1B	109.5	H9A—C9—H9B	108.0
S1—C1—H1C	109.5	S8—C10—H10A	109.5
H1A—C1—H1C	109.5	S8—C10—H10B	109.5
H1B—C1—H1C	109.5	H10A—C10—H10B	109.5
S2—C2—H2A	109.5	S8—C10—H10C	109.5
S2—C2—H2B	109.5	H10A—C10—H10C	109.5
H2A—C2—H2B	109.5	H10B—C10—H10C	109.5
S2—C2—H2C	109.5	C12—C11—C15	117.1 (3)
H2A—C2—H2C	109.5	C12—C11—C9	120.7 (3)
H2B—C2—H2C	109.5	C15—C11—C9	122.2 (3)
C4—C3—S1	123.6 (2)	C11—C12—C13	120.3 (3)
C4—C3—S3	116.7 (2)	C11—C12—H12	119.9
S1—C3—S3	119.55 (18)	C13—C12—H12	119.9
C3—C4—S2	124.6 (2)	N1—C13—C12	122.5 (3)
C3—C4—S4	117.5 (2)	N1—C13—C13^i^	116.2 (3)
S2—C4—S4	117.70 (18)	C12—C13—C13^i^	121.3 (4)
C6—C5—S4	124.5 (2)	N1—C14—C15	124.1 (3)
C6—C5—S3	121.8 (2)	N1—C14—H14	118.0
S4—C5—S3	113.67 (18)	C15—C14—H14	118.0
C5—C6—S5	124.6 (2)	C14—C15—C11	119.3 (3)
C5—C6—S6	121.5 (2)	C14—C15—H15	120.3
S5—C6—S6	113.88 (18)	C11—C15—H15	120.3
C8—C7—S7	125.6 (2)	C14—N1—C13	116.8 (3)
C8—C7—S5	117.1 (2)	C3—S1—C1	104.34 (16)
S7—C7—S5	117.26 (17)	C4—S2—C2	101.90 (17)
C7—C8—S8	124.6 (2)	C3—S3—C5	94.78 (15)
C7—C8—S6	117.1 (2)	C5—S4—C4	94.69 (15)
S8—C8—S6	118.20 (17)	C6—S5—C7	95.17 (14)
C11—C9—S7	111.1 (2)	C8—S6—C6	95.04 (15)
C11—C9—H9A	109.4	C7—S7—C9	99.50 (14)
S7—C9—H9A	109.4	C8—S8—C10	102.28 (16)

Symmetry codes: (i) −*x*+1, −*y*+1, −*z*+1.

## References

[bb1] Altomare, A., Cascarano, G., Giacovazzo, C. & Guagliardi, A. (1993). *J. Appl. Cryst.* **26**, 343–350.

[bb2] Blanchard, P., Dugauy, G., Cousseau, J., Sallé, M., Jubault, M., Gorgues, A., Boubekeur, K. & Batail, P. (1993). *Synth. Met.* **55–57**, 2113–2117.

[bb3] Blessing, R. H. (1995). *Acta Cryst.* A**51**, 33–38. 10.1107/s0108767394005726 7702794

[bb4] Boudiba, L., Gouasmia, A. K., Kaboub, L., Cador, O., Ouahab, L. & Fabre, J.-M. (2005). *Synth. Met.* **150**, 317–320.

[bb5] Burnett, M. N. & Johnson, C. K. (1996). *ORTEPIII* Report ORNL-6895. Oak Ridge National Laboratory, Tennessee, USA.

[bb6] Doria, G., Passarotti, C., Sala, R., Magrini, R., Sberze, P., Tibolla, M., Ceserani, R. & Castello, R. (1986). *Farm. Ed. Sci.* **41**, 417–428. 3743739

[bb7] Fabre, J.-M. (2002). *J. Solid State Chem.* **168**, 367–383.

[bb8] Farrugia, L. J. (1997). *J. Appl. Cryst.* **30**, 565.

[bb9] Farrugia, L. J. (1999). *J. Appl. Cryst.* **32**, 837–838.

[bb11] Gonzalez, A., Segura, J. L. & Martin, N. (2000). *Tetrahedron Lett.* **41**, 3083–3086.

[bb12] Hudhomme, P., Sallé, M., Gautier, N., Belyasmine, A. & Gorgues, A. (2006). *Arkoivoc, ***iv**, 49–72.

[bb13] Ikeda, K., Kawabata, K., Tanaka, K. & Mizutani, M. (1993). *Synth. Met.* **55–57**, 2007–2012.

[bb14] Liu, H.-X., Dolder, S., Franz, P., Neels, A., Stoeckli-Evans, H. & Decurtins, S. (2003). *Inorg. Chem.* **42**, 4801–4803. 10.1021/ic034348h 12895099

[bb15] Oxford Diffraction (2006). *CrysAlis CCD* and *CrysAlis RED* Oxford Diffraction Ltd, Abingdon, Oxfordshire, England.

[bb16] Sakata, J. I., Sato, H., Misayaki, A., Enoki, T., Okano, Y. & Kato, R. (1998). *Solid State Commun.* **108**, 377–381.

[bb17] Setifi, F., Ouahab, L., Gohlen, S., Yoshida, Y. & Saito, G. (2003). *Inorg. Chem.* **42**, 1791–1793. 10.1021/ic026211h 12639109

[bb18] Sheldrick, G. M. (2008). *Acta Cryst.* A**64**, 112–122. 10.1107/S0108767307043930 18156677

[bb19] Watkin, D. M., Pearce, L. & Prout, C. K. A. (1993). *CAMERON* University of Oxford, England.

[bb20] Williams, J. M., Ferraro, J. R., Thorn, R. I., Carlson, K. D., Geiser, U., Wang, H. H. A. M., Kini, A. M. & Wangbo, M. H. (1992). *Organic Superconductors (including Fullerenes), Synthesis, Structure, Properties and Theory* Englewood Cliffs, NJ: Prentice Hall.

[bb21] Yagubskii, E. B. (1993). *Mol. Cryst. Liq. Cryst.* **230**, 139–156.

